# United Kingdom Co-ordinating Committee on Cancer Research (UKCCCR) Strategy Group Workshop

**DOI:** 10.1054/bjoc.2000.1246

**Published:** 2000-07-03


British Journal of Cancer (2000) 83(3), 294–297
© 2000 Cancer Research Campaign
doi: 10.1054/ bjoc.2000.1246, available online at http://www.idealibrary.com on IDEAL®

REPORT

### United Kingdom Co-ordinating Committee on Cancer Research (UKCCCR) Strategy Group Workshop

### 'Clinical endpoints in trials of biological agents'

The Strategy Group of the UKCCCR held a workshop on 18 November, 1999 to consider the issue of 'Clinical endpoints in trials of biological agents'. The organizers of the workshop were Professor Barry Hancock (Chairman, UKCCCR Renal Cancer Group) and Dr Peter Twentyman (Executive Secretary, UKCCCR). The workshop was Chaired by Professor Sir William Asscher (Chairman, UKCCCR) and was attended by representatives of the Cancer Research Campaign (CRC), Imperial Cancer Research Fund (ICRF), Leukaemia Research Fund (LRF) and Medical Research Council (MRC), together with a number of invited speakers and rapporteurs.

In his introductory remarks, the Chairman pointed out that many new types of agent of interest to cancer therapists could not be assessed by the same criteria as conventional cytotoxics. This presents a variety of problems for pharmaceutical companies, clinicians and regulatory agencies in designing appropriate trials and establishing valid endpoints. In doing so, the ultimate goal of patient benefit must always be the guiding principle.

### BACKGROUND TO THE TOPIC

Professor Barry Hancock (University of Sheffield) illustrated some of the problems by referring to the Aim High trial of alpha interferon as adjuvant treatment for high risk melanoma. There are many different types of interferons (both natural and synthetic) and the dose levels being investigated are clearly superphysiological. Although such agents are most likely to work in the adjuvant setting, traditional, early-stage clinical testing relies on the use of patients with 'end-stage' tumours. Dose-response and dose-toxicity relationships may be complicated and the use of pharmacokinetic data may not be possible or helpful. Problems such as these would be more specifically highlighted in the presentations to follow.

### DELIVERY, PHARMACOKINETICS, PHARMACODYNAMICS OF BIOLOGICAL AGENTS – GENERAL ISSUES

Professor Jim Carmichael (University of Nottingham) outlined the standard progression for cytotoxics through phase I→II→III often including pharmacokinetic and pharmacodynamic analysis. However, with newer types of agents, very low doses were often used, and both tumour response and toxicity were liable to be unpredictable. In phase I, it would be difficult to design a schedule of administration, the toxicity profile may be very unusual and it may be hard to define 'evidence of effect'. Some biological agents may be best administered with prolonged low-dose exposure, may have bell-shaped dose-response curves and may have an indirect anti-tumour effect. For agents with specific gene targets it may be possible to incorporate measurements of gene expression into phase I and II but the question of tissue-specificity may be problematic. It may be particularly difficult to agree on what constitutes sufficient evidence to justify progression to phase III development.

Dr Julie Sylvester (Drug Development Office, Cancer Research Campaign) discussed whether the general development strategy for biological agents should be the same as that for cytotoxics. She felt that pharmacodynamic endpoints could be particularly useful for biologicals. In trials of such agents, any novel approaches, including the use of surrogate endpoints would require careful validation. Issues raised by GMP would also need to be borne in mind.

In the discussion, there was much interest in the desirability of measuring interaction between an agent and its intended target rather than an overemphasis on pharmacokinetics. A number of possibilities were presented by the neoadjuvant situation where tumour tissue could be available at surgery following drug administration. Pharmacokinetic measurements were usually only made of plasma concentration whereas the drug concentration in the tumour was the most relevant determinant of efficacy. Professor Alaisdair Breckenridge (Chairman, Committee on Safety of Medicines) emphasized that drug regulation follows science and that applicants must decide whether pharmacokinetic data would strengthen any particular case.

### CYTOKINES

Dr Tim Eisen (University College London) referred to cytokines as 'soluble proteins which act as messengers between cells across the extracellular environment'. Current examples include interferons, interleukins, tumour necrosis factor and colony stimulating factors. They are characterized by pleiotropy, redundancy, and activity in a wide range of tissues. In cancer therapy they may be used alone, or in combination with cytotoxics or other cytokines. In terms of response markers, it is necessary to determine what type of sample should be taken from what site and how it should be processed. A variety of downstream events could potentially be examined from initial effects on kinase activity to clinical phenomena such as disrupted blood flow and cellular apoptosis. In clinical trials it is important to remember that the most effective dose may not be the highest dose and that delayed responses may occur. Unusual side-effects such as vascular leak and delayed auto-immune disease may occur.

Professor Nick Thatcher (University of Manchester) was particularly interested in cytokines as 'supportive agents' for reduction in toxicity of chemotherapy. Endpoints such as 'days spent in hospital' become relevant in such a situation. Cytokines such as interferons and interleukins are interesting because they have some activity in tumour types resistant to conventional chemotherapy. However, the high cost of such agents dictates that health economic analysis will be an essential element of their clinical development. Clearly, in carrying out such analysis, it is important that the right tumour type is studied.

The discussion focused largely on issues relating to combined use of cytokines and cytotoxics. Evidence that each element of such combinations contributes to overall efficiency is often lacking. The presence of vascular leak syndrome at the time of cytotoxic administration could be a major hazard. There was also a problem, where cytokines may produce a delayed tumour response, in that administration may be stopped early on the grounds of early progression.

### VACCINES

Dr Lindy Durrant (University of Nottingham) presented an analysis of tumour vaccines and their mode of action. Clearly if vaccine toxicity is related to expression of a specific antigen then knowledge of the distribution of the antigen on normal tissue is an essential prerequisite. For example, an anti-melanoma vaccine may produce vitiligo by action on melanin-containing normal cells. Tumour tissue can ‘switch off’ both naive and memory T cells and therefore it is better to treat minimal residual disease and immunize aggressively. Phase I trials of vaccines have usually started in advanced disease and progressed to minimal residual disease with schedules/doses being modified to optimize the immune response. Immune monitoring may be carried out on blood samples using a variety of assays or on tumour samples by determination of immune infiltrate. As an example, Dr Durrant quoted their own trial of 105AD7 in colorectal cancer in which patients were immunized prior to operation and boosted post-operatively. Presence of lymphocyte infiltration (CD4, CD8, CD56 and CD68) together with tumour cell apoptosis had been measured in the operative specimen. In all clinical trials of cancer vaccines, measurement of immune response should be regarded as a vital endpoint.

Professor Angus Dalgleish (St. George’s Hospital, London) highlighted a variety of approaches which has been taken in studies which he had carried out. He emphasized that the genetic instability of tumour cells and the presence of heterogeneous clones within tumours presented problems with respect to use of strategies based on single antigens. He showed clinical data for response of melanoma to the ‘Morton vaccine’ based on multiple cell lines, and targeting multiple antigens/epitopes. There had been good correlation between the (ΔCDC) and disease-free survival in patients receiving the vaccine.

In discussion, there was much interest in the need to obtain information on immune effects occurring at the tumour site. Professor Thatcher thought that it should be relatively straightforward to administer a vaccine to patients with progressive disease and subsequently biopsy responding (and non-responding) metastases. It was felt that the problem of tumour heterogeneity may hinder interpretation. However, in general, responding nodules following vaccine therapy are generally seen to have a large lymphocyte infiltrate. It was not considered possible to judge which patients were likely to have a good immune response and this did not depend solely upon bulk of disease. The group considered whether a phase I study in the neoadjuvant setting would allow comprehensive study of tumour effects in the resection specimen. However, it was agreed that good vaccines which produced a delayed effect may be lost via this route. Although measurement of apoptosis in tumour specimens was a potentially useful endpoint, the timing of measurements was a difficult and potentially misleading issue.

### GENERAL DISCUSSION

At the end of the morning session there was a General Discussion. Professor Carmichael was interested in knowing, for various types of agents, what information, at the end of the Phase I stage, would incline investigators to proceed to Phase III. Professor Breckenridge made the point that the Committee on Safety of Medicines now deals with oncology products in a similar manner to other drugs, and similar types of evidence would be required. Professor Dalgleish agreed that it would be unfair to present data only from ‘responding patients’ and that biological response should be assessed on an ‘intention to treat’ basis unless there had been a prior, marker-defined, group in which differential results had occurred. Concern was expressed however that a potentially useful agent, very effective in a subgroup of patients may be lost when decisions are based on results for the whole population. There was major concern expressed that the important effects of a novel agent may be missed if Phase II trials were carried out using an inappropriate schedule. Data to address this concern are not generally available and it would be difficult to obtain academic funding for (e.g.) detailed comparisons of schedules.

### ANTIBODY-DIRECTED THERAPY

The use of antibody-directed therapy was addressed by Professor Richard Begent (Royal Free Hospital, London). He began by outlining the scientific basis of ADEPT (antibody directed enzyme prodrug therapy) and pointed out the need to determine that each component of the system was operative. This would include measurement of enzyme activity in tumour and blood in order that the prodrug can be administered when the enzyme ratio is very high. If necessary, methods may be used to clear artificially the enzyme from the circulation. The relative concentrations of prodrug versus drug in the plasma can then be ascertained and various assays used to detect drug-induced lesions in the tumour cells. Heterogeneity of lesion induction through the tumour may possibly be demonstrated by immunohistochemistry. Using this type of approach a number of responses have been seen in Phase I studies. Progress beyond this will depend upon optimization of parameters in the compartmental model using numeric values from clinical experience. This could include: (a) affinity/antigen and antibody concentrations, (b) flow through tumour extravascular space, (c) elimination of target molecule from tumour, (d) construction of more stable molecule with improved tumour/normal tissue ratio.

Professor Terry Hamblin (University of Southampton) believed that the optimal approach in such studies should rely upon a relatively small number of patients who are very intensively investigated. Novel approaches to Phase I/II testing are likely to be required in situations where increased dose may not lead to increased toxicity. Phase I testing often occurs in patients with a high tumour load and this may act as a ‘sump’ for antibody resulting in a very high MTD. However, more problematic cross-reactivity may occur in patients with lower tumour loads. Hence, choosing the ‘correct dose’ for Phase II may be difficult.

In discussion it was pointed out that, whereas the administered dose for conventional cytotoxics was likely to be determined by toxicity, quite different considerations (e.g. availability, cost)

© 2000 Cancer Research Campaign would prevail for antibodies. There were mixed views on whether investigations of therapeutic efficacy should concentrate on specific cellular targets or more general cellular effects. Not all effects of antibody-directed therapies would necessarily occur via interaction with target antigen. The question of when was the appropriate time to leave a succession of Phase I studies and proceed to Phase II/III was difficult.

### ANTIANGIOGENIC AGENTS

Dr Trivadi Ganesan (University of Oxford) discussed agents which acted as inhibitors of neoangiogenesis, an essential element of tumour growth. Such agents could act via a variety of pathways and include, (a) drugs which inhibit matrix breakdown, (b) drugs which directly inhibit endothelial cell activity, (c) drugs which inhibit activators of angiogenesis, (d) drugs that inhibit endothelial specific integrin/survival signalling, (e) drugs with nonspecific mechanisms. Conventional criteria for MTD were often not appropriate in clinical trials of such agents and surrogate biological endpoints may be more useful. These could include, (a) target specific assays, (b) vascular markers, (c) conventional tumour markers, (d) PET scans, (e) indirect evidence of tumour effects. Specific problems with antiangiogenic agents include their ineffectiveness in bulky disease and the difficulty of combining them with other modalities in an overall plan of cancer management. Side effect profiles may be very unusual as essential physiological functions may be affected.

Professor Ian Hart (St. Thomas' Hospital, London) believed that targeting angiogenesis had a number of clear theoretical advantages. Firstly, as normal cells were the target, development of cellular resistance was unlikely to occur. There was a potential amplification effect in that destruction of a small number of endothelial cells may be expected to eliminate large numbers of tumour cells. Furthermore, the general approach was applicable to all types of solid tumours – irrespective of their tissue of origin or histological type. However, there were, in fact, few data available to support any of these contentions. A very large number of potentially antiangiogenic agents are available but there are few indicators of how they should be prioritized for clinical testing. The relevance of various laboratory assays remains unclear and antitumour testing in animals has frequently employed model systems with fundamental differences from human tumours. Professor Hart believed that the biology is still poorly understood and this makes it difficult to know when and how clinical trials should proceed. Current trials have shown disappointing efficacy despite promising preclinical data.

In discussion, the need for fairly pragmatic trials was emphasized. Approaches could include combination with conventional chemotherapy and in the 'minimal residual disease' situation post surgery. It was agreed that some clinical trials had apparently shown effects greater than could be predicted from a purely cytostatic effect following inhibition of neo angiogenesis. Such results raised questions regarding the mechanism of action of such agents.

### REGULATORY AND LICENSING ISSUES

Professor David Linch (University College, London) discussed how regulatory/licensing issues could impact upon the development of agents and the design of clinical trials. In the case of the use of CSFs as supportive agents, primary endpoints such as 'febrile neutropenia' and 'microbiologically documented infections during neutropenia' had been used after discussion with the regulatory authorities. These endpoints are largely spurious, however, and often fail to reflect the duration of a septic episode. Pharmaco-economic data such as 'time in hospital' might be more clinically relevant but it is a 'soft' end-point not favoured by regulatory authorities. It is also clear that the regulatory authorities demand differing endpoints at different stages of a disease. In lymphoma, at presentation, for example, survival data from randomized trials is required but in relapsed disease, response data may be sufficient for licensing. In the USA redefinition of the 'Phase III trial' has allowed the use of historical cohorts. Finally, it must be noted that there is a long tradition in oncology of using agents for non-licensed indications and the major use for some cytotoxic agents has been outside of these indications.

Professor Alaisdair Breckenridge (Chairman, Committee on Safety of Medicines) drew parallels between licensing of anticancer drugs and other types of agents. These have recently become more similar as cancer agents have moved on from general cytotoxics to drugs with specific targets. Licensing depends upon assessment of efficacy and risk/benefit analysis. He believed there to be good arguments for 'early licensing' of cancer drugs and this would be discussed at a forthcoming CPMO conference. Surrogate markers were becoming widely used as endpoints but this was only valid where a clear correlation with clinical endpoints has been previously established. Use of historical controls was not automatically unacceptable but should be viewed with extreme caution. In general, regulatory criteria follow science and not vice versa!

The use of 'time to progression' as a tumour response endpoint was discussed, particularly in respect of drug licensing. The cancer experts on the CSM were believed to be not unhappy with the concept provided that it was properly defined. It was agreed that 'time to progression' would not, on its own, be a sufficient basis for licensing. The question of early versus late licensing is always difficult but there is, at least, in cancer not the problem of drug use off-indication by GPs. It was agreed that a common misconception is that licensing is, in some way, a 'recommendation for use'. Increasingly the question of 'cost-effectiveness' for cancer drugs arises and it is this precise issue which will be addressed by the National Institute for Clinical Excellence. It was pointed out that companies are often granted a 'conditional registration' for the duration of an ongoing phase III trial.

### ETHICS AND FUNDING ISSUES

Dr Brian Scott (Chairman, Trent MREC) emphasized that the scientific validity of the endpoints used would feature amongst the issues that an ethics committee would consider in deciding approval for a clinical trial. This may be more relevant than the basic aims and design of a trial which, coming from bodies such as the MRC, would be expected to be acceptable. There was however, a strong feeling amongst most of those present that it was inappropriate for ethics committees to judge the scientific merit of clinical projects other than in respect of ethical issues.

Dr John Toy (ICRF) pointed out the patients frequently entered trials when they are at their most vulnerable because they are often at a stage of their illness when all other treatments have failed. Explanation of surrogate endpoints at this time, in such a way that their fully informed consent is validly obtained, may be difficult. One example would be patients being asked to enter neo-adjuvant studies when surgery is to be delayed in the placebo arm, in order

 © 2000 Cancer Research Campaign that its timing matches that in the active biological therapeutic arm. Another difficulty is obtaining fully informed consent for the later and presently unknown analyses of tissue sample donations which are to be stored. A study has shown that the patients who are prepared to enter into randomized trials are increased in numbers when a fuller explanation is given to them about the concept of randomization. Another study, however, has shown that although doctors are willing to enter patients into studies, difficulties of time and energy required to identify eligible patients and to obtain their consent results in fewer than 50% of eligible patients being entered into trials. An ethical role must be allowed for non-medically qualified clinical research staff to help with these activities.

Funding issues predominantly concern the adequacy of providing a trials infrastructure, registries and databases within the NHS. These are topics which have recently been identified by NHS R&D Priorities Working Group as of highest priorities.

### FINAL DISCUSSION

The final discussion highlighted a number of points which had not been mentioned previously. Dr Matt Seymour (Leeds) felt that even small, early phase, trials could benefit from the use of randomization rather than historical controls. However, the problems of acceptability to patients of a ‘no active treatment’ arm should not be underestimated. With respect to a phase II trial, if this is intended as a preliminary to a large phase III, then randomization is important. If, however, the phase II is expected to further early development, then randomization is less essential. Finally, Professor M Dowsett (Institute of Cancer Research) pointed out that a molecular biologist may often set up an assay without knowledge of the degree of variability which will be found in clinical samples. Optional use of surrogate endpoints will require good information regarding such variability.

© 2000 Cancer Research Campaign

